# *trans*-4,5-Dihydr­oxy-1,3-diphenyl­imidazolidine-2-thione

**DOI:** 10.1107/S160053680903548X

**Published:** 2009-09-09

**Authors:** Zhenfeng Zhang, Meilin Wei, Jiange Wang, Guisheng Zhang

**Affiliations:** a College of Chemistry and Environmental Science, Henan Normal University, Xinxiang 453007, People’s Republic of China; b College of Chemistry, Luoyang Normal University, Xinxiang 453007, People’s Republic of China

## Abstract

In the title compound, C_15_H_14_N_2_O_2_S, the five-membered ring adopts an envelope conformation and the two hydr­oxy groups lie on opposite sides of the ring. The six-membered rings are oriented at a dihedral angle of 22.63 (3)°. In the crystal structure, inter­molecular O—H⋯S and O—H⋯O hydrogen bonds link the mol­ecules into a two-dimensional network.

## Related literature

For the biological activity of imidazolidine-2-one derivatives, see: Lam *et al.* (1994 ▶); Lenzen & Ahmad (2001 ▶); Perronnet & Teche (1973 ▶). For related structures, see: Enders *et al.* (1979 ▶); Zhang *et al.* (2007 ▶). 
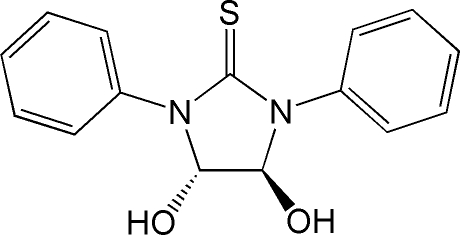


## Experimental

### 

#### Crystal data

C_15_H_14_N_2_O_2_S
*M* *_r_* = 286.34
Orthorhombic, 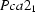

*a* = 20.5119 (4) Å
*b* = 7.1020 (4) Å
*c* = 9.6659 (3) Å
*V* = 1408.09 (9) Å^3^
*Z* = 4
Mo *K*α radiation
μ = 0.23 mm^−1^
*T* = 294 K
0.35 × 0.22 × 0.20 mm

#### Data collection

Bruker SMART CCD area-detector diffractometer
Absorption correction: multi-scan (*SADABS*; Sheldrick, 2003 ▶) *T*_min_ = 0.923, *T*_max_ = 0.944
7542 measured reflections
2521 independent reflections
2273 reflections with *I* > 2σ(*I*)
*R*_int_ = 0.045

#### Refinement

*R*[*F* ^2^ > 2σ(*F* ^2^)] = 0.037
*wR*(*F* ^2^) = 0.119
*S* = 1.09
2521 reflections
183 parameters
H-atom parameters constrained
Δρ_max_ = 0.26 e Å^−3^
Δρ_min_ = −0.40 e Å^−3^
Absolute structure: Flack (1983 ▶), 1123 Friedel pairs
Flack parameter: 0.16 (10)

### 

Data collection: *SMART* (Bruker, 1997 ▶); cell refinement: *SAINT* (Bruker, 1997 ▶); data reduction: *SAINT*; program(s) used to solve structure: *SHELXS97* (Sheldrick, 2008 ▶); program(s) used to refine structure: *SHELXL97* (Sheldrick, 2008 ▶); molecular graphics: *SHELXTL* (Sheldrick, 2008 ▶); software used to prepare material for publication: *SHELXTL*.

## Supplementary Material

Crystal structure: contains datablocks I, global. DOI: 10.1107/S160053680903548X/hk2762sup1.cif

Structure factors: contains datablocks I. DOI: 10.1107/S160053680903548X/hk2762Isup2.hkl

Additional supplementary materials:  crystallographic information; 3D view; checkCIF report

## Figures and Tables

**Table 1:** Hydrogen-bond geometry (Å, °)

*D*—H⋯*A*	*D*—H	H⋯*A*	*D*⋯*A*	*D*—H⋯*A*
O1—H1⋯S1^i^	0.91	2.41	3.261 (2)	156
O2—H2⋯O1^ii^	0.82	2.13	2.930 (3)	167

Symmetry codes: (i) 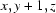; (ii) 
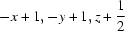
.

**Fig. 1.**
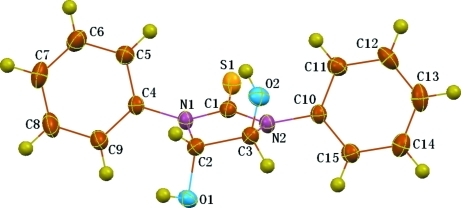
The molecular structure of the title molecule, with the atom-numbering scheme. Displacement ellipsoids are drawn at the 30% probability level.

**Fig. 2.**
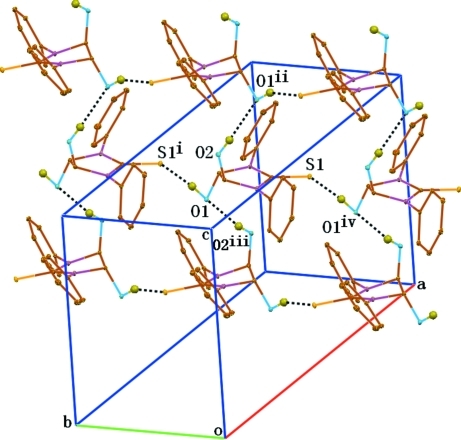
A partial packing diagram of the title compound. For the sake of clarity, H atoms not involved in hydrogen bonding have been omitted. Hydrogen bonds are shown as dashed lines. Selected atoms are labelled. [Symmetry codes: (i) x, y + 1, z; (ii) -x + 1, -y + 1, z + 1/2; (iii) -x + 1, -y + 1, z - 1/2; (iv) x, y - 1, z].

## supplementary crystallographic information

### Comment

Imidazolidine-2-one derivates often exhibit powerful bioactivities, such as good herbicidal activity (Perronnet & Teche,1973), antidiabetic properties (Lenzen & Ahmad, 2001) and anti-HIV activity (Lam *et al.*, 1994). Enders *et al.* (1979) have earlier reported the synthesis and use of 4,5-dihydroxyimidazolidine-2-thiones. However, to the best of our knowledge, there are few *N*,*N*'-diaryl substituted 4,5-dihydroxyimidazolidine-2 -thiones reported so far. As a typical example of such compounds, we report herein the crystal structure of the title compound.

In the molecule of the title compound, (I), (Fig. 1) the five-membered ring A (N1/N2/C1-C3) adopts an envelope conformation with atom C3 displaced by -0.369 (3) Å from the plane of the other ring atoms. The two hydroxyl groups lie on opposite sides of the ring. The C1-N1 [1.357 (3) Å] and C1-N2 [1.373 (3) Å] bonds are longer than the corresponding bonds in trans-4,5-dihydroxyimidazolidine-2-thione, (II), [1.335 (2) and 1.336 (2) Å; Zhang *et al.*, 2007]. Conversely, the C1═S1 [1.669 (3) Å] and C2-C3 [1.526 (4) Å] bonds in (I) are shorter than the corresponding bonds in (II) [1.684 (2) and 1.537 (2) Å, respectively]. Rings B (C4-C9) and C (C10-C15) are, of course, planar and they are oriented at a dihedral angle of B/C = 22.63 (3)°.

In the crystal structure, intermolecular O-H···S A and O-H···O hydrogen bonds (Table 1) link the molecules into a two-dimensional network (Fig. 2), in which they may be effective in the stabilization of the structure.

### Experimental

For the preparation of the title compound, 1,3-diphenylthiourea (0.1 mol), glyoxal (40%, 18 g) and ethanol (95%, 30 ml) were added into a three-necked round-bottomed flask equipped with a stirrer. The mixture was then refluxed with stirring for *ca* 30 min and thereafter the solvent was removed. The residue was washed with cold ethanol, and the resulting solid product was recrystallized from hot ethanol to give the crystals of the title compound. ^1^H NMR(DMSO, 400 MHz) of (I): δ 7.52–7.28 (m, 10H), δ 7.10 (d, J = 8.0 Hz, 2H), δ 5.19 (d, J = 8.4 Hz, 2H).

### Refinement

Atom H1 (for OH) is located in a difference Fourier map and only its temperature factor is refined. The remaining H atoms were positioned geometrically with O-H = 0.82 Å (for OH) and C-H = 0.93 and 0.98 Å for aromatic and methine H atoms, respectively, and constrained to ride on their parent atoms, with U_iso_(H) = xU_eq_(C,O), where x = 1.5 for OH H and x = 1.2 for all other H atoms.

### Crystal data

**Table:**

C_15_H_14_N_2_O_2_S	*F*(000) = 600
*M**_r_* = 286.34	*D*_x_ = 1.351 Mg m^−^^3^
Orthorhombic, *P**c**a*2_1_	Mo *K*α radiation, λ = 0.71073 Å
Hall symbol: P 2c -2ac	Cell parameters from 871 reflections
*a* = 20.5119 (4) Å	θ = 2.9–27.9°
*b* = 7.1020 (4) Å	µ = 0.23 mm^−^^1^
*c* = 9.6659 (3) Å	*T* = 294 K
*V* = 1408.09 (9) Å^3^	Block, colorless
*Z* = 4	0.35 × 0.22 × 0.20 mm

### Data collection

**Table:**

Bruker SMART CCD area-detector diffractometer	2521 independent reflections
Radiation source: fine-focus sealed tube	2273 reflections with *I* > 2σ(*I*)
graphite	*R*_int_ = 0.045
φ and ω scans	θ_max_ = 25.5°, θ_min_ = 2.9°
Absorption correction: multi-scan (*SADABS*; Sheldrick, 2003)	*h* = −24→24
*T*_min_ = 0.923, *T*_max_ = 0.944	*k* = −8→8
7542 measured reflections	*l* = −11→11

### Refinement

**Table:**

Refinement on *F*^2^	Secondary atom site location: difference Fourier map
Least-squares matrix: full	Hydrogen site location: inferred from neighbouring sites
*R*[*F*^2^ > 2σ(*F*^2^)] = 0.037	H-atom parameters constrained
*wR*(*F*^2^) = 0.119	*w* = 1/[σ^2^(*F*_o_^2^) + (0.0758*P*)^2^ + 0.1052*P*] where *P* = (*F*_o_^2^ + 2*F*_c_^2^)/3
*S* = 1.09	(Δ/σ)_max_ = 0.001
2521 reflections	Δρ_max_ = 0.26 e Å^−^^3^
183 parameters	Δρ_min_ = −0.40 e Å^−^^3^
0 restraints	Absolute structure: Flack (1983), 1123 Friedel pairs
Primary atom site location: structure-invariant direct methods	Flack parameter: 0.16 (10)

### Special details

**Table:**

Geometry. All e.s.d.'s (except the e.s.d. in the dihedral angle between two l.s. planes) are estimated using the full covariance matrix. The cell e.s.d.'s are taken into account individually in the estimation of e.s.d.'s in distances, angles and torsion angles; correlations between e.s.d.'s in cell parameters are only used when they are defined by crystal symmetry. An approximate (isotropic) treatment of cell e.s.d.'s is used for estimating e.s.d.'s involving l.s. planes.
Refinement. Refinement of *F*^2^ against ALL reflections. The weighted *R*-factor *wR* and goodness of fit *S* are based on *F*^2^, conventional *R*-factors *R* are based on *F*, with *F* set to zero for negative *F*^2^. The threshold expression of *F*^2^ > σ(*F*^2^) is used only for calculating *R*-factors(gt) *etc*. and is not relevant to the choice of reflections for refinement. *R*-factors based on *F*^2^ are statistically about twice as large as those based on *F*, and *R*- factors based on ALL data will be even larger.

### Fractional atomic coordinates and isotropic or equivalent isotropic displacement parameters (Å^2^)

**Table:**

	*x*	*y*	*z*	*U*_iso_*/*U*_eq_	
S1	0.57085 (3)	0.08292 (10)	0.82543 (8)	0.0490 (2)	
O1	0.50025 (9)	0.6924 (3)	0.73031 (18)	0.0461 (5)	
H1	0.5111	0.7982	0.7786	0.16 (3)*	
O2	0.41663 (9)	0.4806 (3)	1.01911 (19)	0.0496 (5)	
H2	0.4449	0.4376	1.0700	0.074*	
N1	0.55261 (10)	0.4549 (3)	0.8636 (2)	0.0377 (5)	
N2	0.46089 (9)	0.2969 (3)	0.8350 (2)	0.0390 (5)	
C1	0.52752 (11)	0.2806 (4)	0.8419 (2)	0.0377 (5)	
C2	0.50147 (11)	0.5976 (4)	0.8604 (2)	0.0369 (6)	
H2A	0.5071	0.6878	0.9363	0.044*	
C3	0.43958 (12)	0.4818 (4)	0.8810 (2)	0.0380 (6)	
H3	0.4053	0.5286	0.8194	0.046*	
C4	0.61952 (11)	0.4991 (4)	0.8966 (2)	0.0365 (5)	
C5	0.64923 (14)	0.4175 (4)	1.0118 (3)	0.0488 (7)	
H5	0.6268	0.3293	1.0647	0.059*	
C6	0.71267 (15)	0.4689 (5)	1.0472 (4)	0.0610 (8)	
H6	0.7327	0.4125	1.1229	0.073*	
C7	0.74579 (15)	0.6012 (5)	0.9721 (4)	0.0626 (9)	
H7	0.7880	0.6358	0.9966	0.075*	
C8	0.71551 (16)	0.6831 (6)	0.8587 (3)	0.0703 (10)	
H8	0.7376	0.7737	0.8075	0.084*	
C9	0.65245 (13)	0.6318 (4)	0.8203 (3)	0.0551 (7)	
H9	0.6328	0.6870	0.7437	0.066*	
C10	0.41476 (10)	0.1456 (4)	0.8282 (3)	0.0381 (5)	
C11	0.40282 (15)	0.0370 (5)	0.9455 (3)	0.0505 (7)	
H11	0.4276	0.0542	1.0251	0.061*	
C12	0.35357 (17)	−0.0971 (4)	0.9426 (4)	0.0585 (8)	
H12	0.3456	−0.1715	1.0200	0.070*	
C13	0.31627 (13)	−0.1201 (4)	0.8242 (4)	0.0612 (8)	
H13	0.2824	−0.2071	0.8237	0.073*	
C14	0.32898 (15)	−0.0150 (5)	0.7067 (3)	0.0563 (8)	
H14	0.3046	−0.0337	0.6268	0.068*	
C15	0.37826 (14)	0.1181 (4)	0.7087 (3)	0.0450 (6)	
H15	0.3870	0.1892	0.6301	0.054*	

### Atomic displacement parameters (Å^2^)

**Table:**

	*U*^11^	*U*^22^	*U*^33^	*U*^12^	*U*^13^	*U*^23^
S1	0.0455 (3)	0.0357 (4)	0.0658 (4)	0.0026 (3)	0.0023 (3)	−0.0088 (3)
O1	0.0571 (12)	0.0356 (12)	0.0456 (9)	−0.0020 (8)	0.0007 (7)	0.0041 (8)
O2	0.0486 (10)	0.0555 (14)	0.0447 (9)	0.0059 (10)	0.0092 (8)	0.0014 (9)
N1	0.0353 (10)	0.0304 (12)	0.0473 (11)	−0.0039 (9)	−0.0010 (8)	−0.0025 (8)
N2	0.0370 (10)	0.0310 (12)	0.0489 (9)	−0.0026 (8)	−0.0008 (9)	−0.0005 (10)
C1	0.0369 (11)	0.0410 (15)	0.0353 (10)	−0.0014 (10)	0.0006 (9)	−0.0012 (11)
C2	0.0401 (13)	0.0327 (16)	0.0379 (12)	0.0032 (10)	−0.0019 (8)	−0.0044 (10)
C3	0.0399 (12)	0.0335 (16)	0.0407 (11)	0.0028 (11)	−0.0016 (10)	−0.0030 (10)
C4	0.0378 (12)	0.0293 (14)	0.0423 (11)	−0.0014 (11)	0.0020 (9)	−0.0078 (10)
C5	0.0449 (14)	0.0473 (18)	0.0542 (13)	−0.0027 (12)	−0.0048 (11)	0.0048 (12)
C6	0.0506 (17)	0.062 (2)	0.0708 (19)	0.0008 (15)	−0.0181 (15)	0.0007 (16)
C7	0.0369 (14)	0.072 (2)	0.0792 (19)	−0.0100 (15)	−0.0032 (13)	−0.0167 (17)
C8	0.0579 (17)	0.082 (3)	0.071 (2)	−0.0333 (17)	0.0108 (15)	0.0005 (18)
C9	0.0545 (14)	0.062 (2)	0.0488 (13)	−0.0183 (14)	0.0016 (13)	0.0044 (16)
C10	0.0354 (10)	0.0335 (14)	0.0453 (11)	−0.0013 (9)	0.0018 (10)	−0.0037 (12)
C11	0.0591 (16)	0.0430 (18)	0.0494 (13)	−0.0023 (14)	−0.0004 (13)	0.0042 (13)
C12	0.069 (2)	0.0369 (18)	0.0694 (17)	−0.0075 (14)	0.0181 (15)	0.0062 (15)
C13	0.0464 (14)	0.0455 (19)	0.092 (2)	−0.0086 (12)	0.0099 (16)	−0.0186 (19)
C14	0.0497 (16)	0.054 (2)	0.0654 (18)	−0.0037 (15)	−0.0079 (12)	−0.0150 (15)
C15	0.0498 (15)	0.0362 (16)	0.0490 (13)	−0.0011 (12)	−0.0005 (11)	−0.0015 (12)

### Geometric parameters (Å, °)

**Table:**

S1—C1	1.669 (3)	C6—C7	1.368 (5)
O1—C2	1.427 (3)	C6—H6	0.9300
O1—H1	0.9123	C7—C8	1.387 (5)
O2—C3	1.416 (3)	C7—H7	0.9300
O2—H2	0.8200	C8—C9	1.394 (4)
N1—C1	1.357 (3)	C8—H8	0.9300
N1—C4	1.444 (3)	C9—H9	0.9300
N1—C2	1.459 (3)	C10—C15	1.390 (4)
N2—C1	1.373 (3)	C10—C11	1.393 (4)
N2—C10	1.433 (3)	C11—C12	1.389 (4)
N2—C3	1.454 (3)	C11—H11	0.9300
C2—C3	1.526 (4)	C12—C13	1.386 (6)
C2—H2A	0.9800	C12—H12	0.9300
C3—H3	0.9800	C13—C14	1.384 (6)
C4—C9	1.374 (4)	C13—H13	0.9300
C4—C5	1.396 (4)	C14—C15	1.384 (4)
C5—C6	1.394 (4)	C14—H14	0.9300
C5—H5	0.9300	C15—H15	0.9300
			
C2—O1—H1	86.1	C7—C6—H6	119.6
C3—O2—H2	109.5	C5—C6—H6	119.6
C1—N1—C4	126.4 (2)	C6—C7—C8	119.0 (3)
C1—N1—C2	110.97 (19)	C6—C7—H7	120.5
C4—N1—C2	122.5 (2)	C8—C7—H7	120.5
C1—N2—C10	126.6 (2)	C7—C8—C9	121.1 (3)
C1—N2—C3	111.1 (2)	C7—C8—H8	119.5
C10—N2—C3	119.53 (19)	C9—C8—H8	119.5
N1—C1—N2	108.0 (2)	C4—C9—C8	119.5 (3)
N1—C1—S1	125.46 (17)	C4—C9—H9	120.3
N2—C1—S1	126.6 (2)	C8—C9—H9	120.3
O1—C2—N1	111.04 (18)	C15—C10—C11	120.3 (2)
O1—C2—C3	110.76 (19)	C15—C10—N2	120.0 (2)
N1—C2—C3	102.8 (2)	C11—C10—N2	119.6 (2)
O1—C2—H2A	110.7	C12—C11—C10	119.4 (3)
N1—C2—H2A	110.7	C12—C11—H11	120.3
C3—C2—H2A	110.7	C10—C11—H11	120.3
O2—C3—N2	112.5 (2)	C13—C12—C11	119.9 (3)
O2—C3—C2	113.8 (2)	C13—C12—H12	120.0
N2—C3—C2	101.37 (19)	C11—C12—H12	120.0
O2—C3—H3	109.6	C14—C13—C12	120.7 (3)
N2—C3—H3	109.6	C14—C13—H13	119.7
C2—C3—H3	109.6	C12—C13—H13	119.7
C9—C4—C5	119.9 (2)	C15—C14—C13	119.6 (3)
C9—C4—N1	119.9 (2)	C15—C14—H14	120.2
C5—C4—N1	120.1 (2)	C13—C14—H14	120.2
C6—C5—C4	119.6 (3)	C14—C15—C10	120.1 (3)
C6—C5—H5	120.2	C14—C15—H15	120.0
C4—C5—H5	120.2	C10—C15—H15	120.0
C7—C6—C5	120.9 (3)		
			
C4—N1—C1—N2	170.7 (2)	C1—N1—C4—C5	−56.2 (3)
C2—N1—C1—N2	−4.7 (3)	C2—N1—C4—C5	118.7 (3)
C4—N1—C1—S1	−9.7 (3)	C9—C4—C5—C6	−1.2 (4)
C2—N1—C1—S1	174.86 (17)	N1—C4—C5—C6	−176.5 (3)
C10—N2—C1—N1	−172.8 (2)	C4—C5—C6—C7	1.3 (5)
C3—N2—C1—N1	−11.8 (3)	C5—C6—C7—C8	−0.6 (5)
C10—N2—C1—S1	7.7 (3)	C6—C7—C8—C9	−0.4 (5)
C3—N2—C1—S1	168.61 (19)	C5—C4—C9—C8	0.2 (4)
C1—N1—C2—O1	−100.5 (2)	N1—C4—C9—C8	175.6 (3)
C4—N1—C2—O1	83.9 (2)	C7—C8—C9—C4	0.5 (5)
C1—N1—C2—C3	18.0 (2)	C1—N2—C10—C15	−112.5 (3)
C4—N1—C2—C3	−157.6 (2)	C3—N2—C10—C15	87.9 (3)
C1—N2—C3—O2	−99.8 (2)	C1—N2—C10—C11	72.9 (3)
C10—N2—C3—O2	62.7 (3)	C3—N2—C10—C11	−86.6 (3)
C1—N2—C3—C2	22.0 (2)	C15—C10—C11—C12	−0.8 (4)
C10—N2—C3—C2	−175.5 (2)	N2—C10—C11—C12	173.7 (3)
O1—C2—C3—O2	−143.2 (2)	C10—C11—C12—C13	−0.9 (5)
N1—C2—C3—O2	98.1 (2)	C11—C12—C13—C14	2.2 (5)
O1—C2—C3—N2	95.8 (2)	C12—C13—C14—C15	−1.8 (5)
N1—C2—C3—N2	−22.9 (2)	C13—C14—C15—C10	0.1 (4)
C1—N1—C4—C9	128.4 (3)	C11—C10—C15—C14	1.2 (4)
C2—N1—C4—C9	−56.7 (3)	N2—C10—C15—C14	−173.3 (2)

### Hydrogen-bond geometry (Å, °)

**Table:**

*D*—H···*A*	*D*—H	H···*A*	*D*···*A*	*D*—H···*A*
O1—H1···S1^i^	0.91	2.41	3.261 (2)	156
O2—H2···O1^ii^	0.82	2.13	2.930 (3)	167

Symmetry codes: (i) *x*, *y*+1, *z*; (ii) −*x*+1, −*y*+1, *z*+1/2.

## References

[bb1] Bruker (1997). *SMART* and *SAINT* Bruker AXS Inc., Madison, Wisconsin, USA.

[bb2] Enders, E., Ebbighausen, V., Gau, W., Wunsche, C. & Stendel, W. (1979). US Patent 4 173 645.

[bb3] Flack, H. D. (1983). *Acta Cryst.* A**39**, 876–881.

[bb4] Lam, P. Y. S., Jadhav, P. K., Eyermann, C. J., Hodge, C. N., Ru, Y., Bacheler, L. T., Meek, J. L., Otto, M. J., Rayner, M. M., Wong, Y. N., Chang, C.-H., Weber, P. C., Jackson, D. A., Sharpe, T. R. & Erickson-Viitanen, S. (1994). *Science*, **263**, 380–384. 10.1126/science.8278812 8278812

[bb5] Lenzen, S. & Ahmad, R. (2001). Ger. Offen. DE10012401.

[bb6] Perronnet, J. & Teche, A. (1973). US Patent 3 905 996.

[bb7] Sheldrick, G. M. (2003). *SADABS* University of Göttingen, Germany.

[bb8] Sheldrick, G. M. (2008). *Acta Cryst.* A**64**, 112–122. 10.1107/S0108767307043930 18156677

[bb9] Zhang, Z.-F., Zhang, J.-M., Guo, J.-P. & Qu, G.-R. (2007). *Acta Cryst.* E**63**, o2821–o2823.

